# DRESS Syndrome in the ICU: When a Patient Is Treated with Multiple Drugs

**DOI:** 10.1155/2016/9453286

**Published:** 2016-01-24

**Authors:** Florent Moriceau, Johanne Prothet, Benjamin J. Blaise, Benoit Ben Said, Mathieu Page, Charles-Eric Ber, Jullien Crozon, Thomas Rimmelé

**Affiliations:** ^1^Département d'Anesthésie-Réanimation, Hôpital Edouard Herriot, 5 place d'Arsonval, 69003 Lyon, France; ^2^Département de Néonatologie et de Réanimation Néonatale, Hôpital Femme Mère Enfant, 32 Avenue Doyen Jean Lépine, 69500 Bron, France; ^3^Département d'Allergologie et d'Immunologie Clinique, Centre Hospitalier de Lyon-Sud, 165 Chemin du Grand Revoyet, 69310 Pierre-Bénite, France; ^4^Université Claude Bernard Lyon 1, 43 Boulevard du 11 Novembre 1918, 69100 Villeurbanne, France

## Abstract

The Drug Reaction with Eosinophilia and Systemic Symptoms (DRESS) syndrome is life-threatening. It associates a skin condition with hematological and visceral disorders. The DRESS syndrome diagnosis in the intensive care unit (ICU) is difficult as clinical features are nonspecific. Furthermore, the need to treat patients with multiple drugs usually prevents the identification of the causative drug. We report the case of a patient who developed two bouts of DRESS caused by piperacillin-tazobactam, the first being complicated with a distributive shock. Cases of DRESS occurring inside ICU are seldom reported. However, any intensivist may encounter this situation during his career and should be aware of its diagnostic and management specific aspects.

## 1. Introduction

The DRESS (Drug Reaction with Eosinophilia and Systemic Symptoms) syndrome is a severe drug hypersensitivity reaction. Although it is less known than Lyell or Stevens-Johnson syndromes and less striking than anaphylactic shock, it can result in a dreadful prognosis, with a mortality rate of 2 to 45% [1–3]. DRESS occurring in an intensive care unit (ICU) is a complex situation. It can mimic more usual causes of shock and organ failure, such as sepsis. Furthermore, identifying the responsible treatment may not be straightforward due to the multiple drugs use in the intensive care unit. Finally, the necessity to remove any suspected treatment will make the patient's management more complicated. We report the case of DRESS induced by piperacillin-tazobactam initially identified as a septic shock and reoccurring after the inappropriate reintroduction of a suspected treatment. The patient's family gave a written consent to report this case.

## 2. Case Report

A 53-year-old woman was admitted to the ICU due to angiocholitis complicated by a septic shock. She had a medical history of high blood pressure and, despite two episodes of kidney transplantation, she had recently reached end-stage renal disease. She was under antihypertensive therapy and immunosuppressant drugs (prednisolone, mycophenolate, and cyclosporine).

**Figure 1:**
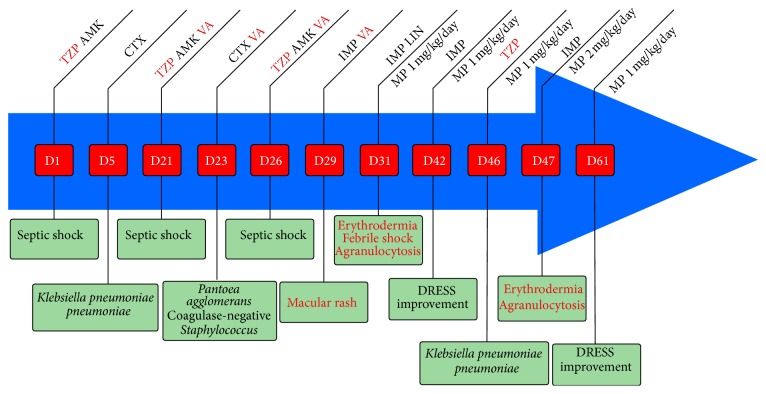
The chronology of events and drugs use related to DRESS occurrences. D: day; TZP: piperacillin-tazobactam; AMK: amikacin; CTX: ceftriaxone; VA: vancomycin; IMP: imipenem; LIN: linezolid; MP: methylprednisolone. Day 1: admission to the ICU.

The early evolution was positive with a probabilistic antibiotherapy associating piperacillin-tazobactam and amikacin. It was decreased to ceftriaxone alone on the fifth day, after the identification of a biliary* Klebsiella pneumoniæ pneumoniæ* (Figure 1). On the 21st day after the ICU admission, the patient developed another septic shock due to angiocholitis, forcing the reintroduction of the initial probabilistic antibiotherapy. On the 23rd day, the antibiotherapy was modified again to ceftriaxone and vancomycin, after the bloodstream identification of a* Pantoea agglomerans* and a coagulase-negative* Staphylococcus*. A third round of septic shock occurred on the 26th day leading us to widen the antibiotherapy, associating piperacillin-tazobactam and amikacin to the already administered vancomycin (Figure 1).

**Figure 2:**
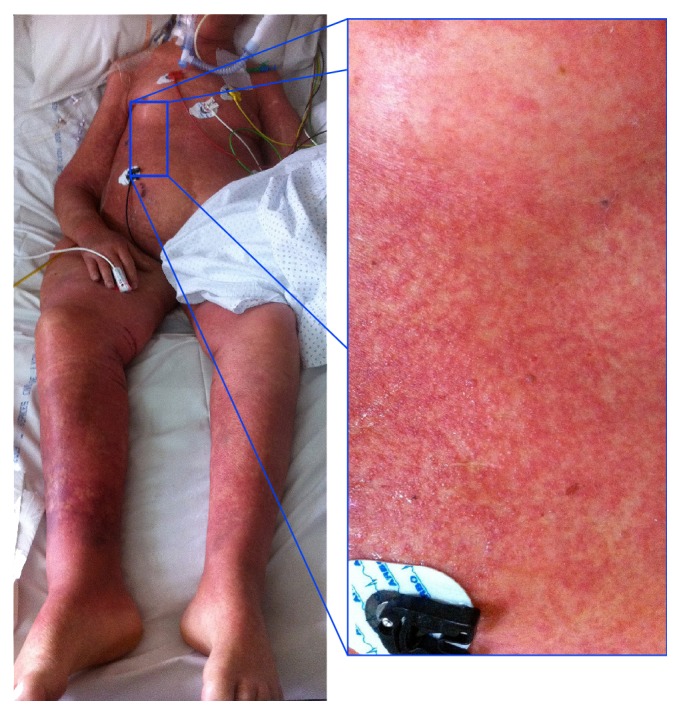
Erythrodermia in ICU ventilated patient. Focus on the chest showing a maculopapular rash.

Following this last modification, a benign-looking rash appeared on the patient's chest on the 29th day. After 48 h of hemodynamic stability, a high fever and a distributive shock requiring consequent norepinephrine infusion (1 *μ*g/kg/min) emerged. The procalcitonin measurement reached a high level (2.98 *μ*g/L), which was compatible with sepsis. However, the rash evolved into erythrodermia (Figure 2), associated with enlarged cervical and inguinal lymph nodes, eosinophilia (absolute eosinophil count of 1.0 × 10^9^/L), agranulocytosis (absolute neutrophil count of 0.4 × 10^9^/L), and activated circulating T-lymphocytes. These elements led to the diagnosis of DRESS syndrome.

All drugs introduced at least ten days before the rash were interrupted, except for vasopressors. Vancomycin was replaced by linezolid. A systemic corticotherapy with methylprednisolone (1 mg/kg/d) was initiated, along with the use of granulocyte colony-stimulating factors (G-CSF).

Eosinophilia reached a maximum of 4.2 × 10^9^/L on the 32nd day, associated with a thrombocytopenia and a nonregenerative anemia. A myelogram revealed a hypoplastic bone marrow, mostly composed of eosinophils (30%) without hemophagocytic lymphohistiocytosis. A skin biopsy showed basal membrane vacuoles, spongiosis, and an inflammatory infiltration of dermis, described as activated lymphocytes and eosinophils. The Epstein-Barr Virus (EBV) load was found elevated at 700 copies/mL. No differential diagnosis was identified.

The cutaneous condition improved within 10 days, from the chest to the limbs, with a reepithelialization following a desquamation and blisters. Eosinophilia and agranulocytosis normalized within five days. However, the nonregenerative anemia and the thrombocytopenia remained.

Vancomycin was initially suspected. Therefore, piperacillin-tazobactam was used again during another bout of sepsis, after the identification of a sensitive* Klebsiella pneumoniæ pneumoniæ* (Figure 1). Erythrodermia reoccurred immediately following this reintroduction, with early eosinophilia (absolute eosinophil count of 1.0 × 10^9^/L), deep agranulocytosis (undetectable neutrophils), and hyperlactatemia (3.2 mmol/L), but without hemodynamic instability. This new DRESS was associated with EBV reactivation (viral load of 5,200 copies/mL). A new myelogram reported a highly hypoplastic bone marrow, with more eosinophils (40%) than previously counted, and the noticeable absence of neutrophilic cells. We thus interrupted the piperacillin-tazobactam administration and increased methylprednisolone up to 2 mg/kg/d. Cutaneous and hematological conditions improved within two weeks. This recurrence clearly incriminated piperacillin-tazobactam.

Later evolution was unfortunately negative, with numerous ICU-associated adverse events (ventilator-associated pneumonia, neuromyopathy, and severe malnutrition). The patient died of septic shock complications triggered by pneumonia, on day 102 after ICU admission.

## 3. Discussion

An occurrence of DRESS inside the ICU is difficult to diagnose, and its management is not obvious. In addition, the intensive care specific aspects are seldom mentioned in the literature.

The DRESS syndrome is a delayed hypersensitivity reaction. Its clinical features include a cutaneous reaction (almost 100% of cases, usually maculopapular, often itchy and extensive to the whole body), a facial edema (76%), a polyadenopathy (54%), a fever (90%), and an organ involvement (91%, either liver, lung, brain, kidney, or heart) [2, 4, 5]. Mortality ranges from 2 to 45% depending on the severity of the organ involvement [1–3, 6]. Few cases of shock have recently been reported, highlighting the need to mention the DRESS syndrome in the diagnosis algorithm of a distributive shock [6]. Hematological abnormalities include a possible eosinophilia (95%) which can be delayed, with either lymphopenia (5%) or lymphocytosis, and often circulating activated T-lymphocytes (67%), as observed in infectious mononucleosis [2]. A hemophagocytic lymphohistiocytosis is not uncommon. It is associated with a worse prognosis and sometimes preceded by biological marker raises (hyperferritinemia, hypertriglyceridemia, and elevated lactate dehydrogenase levels). Procalcitonin can rise regardless of any sepsis [5].

The pathophysiology of the organ involvement is multifactorial and still not fully understood. Human herpes-virus family reactivations, their local proliferation, and the cytotoxic immune response they induce may be involved. Indeed, viral DNAs have been identified in affected organs, but no causal link has yet been established [3, 7, 8]. The viral load is a diagnosis criterion in some countries [9]. Descamps and Ranger-Rogez also reported a genetic predisposition that influences cytotoxic T-lymphocytes response [5].

The list of medications that may potentially induce DRESS keeps growing and includes more than 40 drugs. The most frequent triggers are (in descending order) anticonvulsants (carbamazepine, lamotrigine, phenobarbital, phenytoin, and sodium valproate), allopurinol, psychotropic drugs, sulfonamides (dapsone, sulfasalazine, and sulfamethoxazole), antiviral therapies, and antibiotics (mostly vancomycin and beta-lactam antibiotics) [2, 4, 10, 11]. The DRESS syndrome is a relatively rare drug reaction with an incidence of 1/5000 for anticonvulsants [12]. It occurs with a median delay of 22 days after the administration of the causal treatment [2]. Any drug initiated one to four weeks before the rash must be suspected. In the intensive care unit, the use of multiple medications may prevent the identification of the responsible drug. This is usually achieved after full recovery, through further allergological investigations such as skin or immunobiological tests (lymphocytes proliferation and cytokine production assays). In this case, the antibiotic changes increased the number of potential causal drugs. Vancomycin was the initial main suspect as it is the most common trigger for DRESS among antibiotics [2], and as the 10-day delay between introduction and symptoms was compatible with the pathophysiology of the DRESS. Furthermore, piperacillin-tazobactam had seldom been related to DRESS at that time, with only three cases reported [13–15]. Since then, Cabañas et al. have added 8 more [16]. Interestingly, piperacillin-tazobactam had been used only 24 hours before the last onset of symptoms. The delay of the symptoms onset can indeed be dramatically shortened in the case of a previous exposure, as the sensitized antigen-specific T-lymphocytes can remain activated for years [17, 18]. Thus, we observed a shortened delay and an intensified reaction when DRESS reoccurred.

Ruling out the differential diagnoses is an additional difficulty in the ICU. DRESS can indeed complicate an underlying condition, such as sepsis with which it shares many clinical features. Kardaun et al. [4] suggested a score that classifies cases as definite, probable, possible, or no case. Relevant clinical features were fever, enlarged lymph nodes, a skin condition, and a late resolution (more than 15 days). Additional criteria were hematological abnormalities (eosinophilia, activated circulating lymphocytes), a compatible skin biopsy, and organ involvement. They also considered the unfruitful research of differential diagnoses, after ruling out autoimmune diseases (lupus, Kawasaki disease), infectious diseases (mononucleosis or other viral exanthems, toxic shock syndrome), and hematological diseases (angioimmunoblastic lymphoma, hypereosinophilic syndrome), to be relevant. In the present case, Kardaun's score reached 8 points, making the diagnosis of DRESS syndrome certain.

The key element in the management of a patient with DRESS is the early and permanent withdrawal of every suspected medication. The use of strong topical steroids is sufficient for mild cases. Severe cases will require the use of a systemic corticotherapy (methylprednisolone 1-2 mg/kg/d). In our case, the early corticotherapy initiated with 1 mg/kg/d of methylprednisolone allowed a general improvement but did not prevent the recurrence. Full recovery will generally be obtained after weeks, even with an early and optimal management [5]. Intravenous immunoglobulins are no longer recommended due to their frequent secondary effects and lack of benefit compared to corticotherapy [19]. A granulopoietic stimulant (G-CSF) can be discussed. On one hand, it may shorten the duration of agranulocytosis. On the other hand, it may redirect the immune response of T4 lymphocytes from the hyperactivated T helper 1 (Th-1) response to the Th-2 response [20, 21]. Viral reactivations may benefit from a specific treatment.

## 4. Conclusion

Any intensivist may encounter or may have to manage a patient with DRESS syndrome. In the ICU, clinical features and organ involvement have a poor specificity. The suspicious drugs are often numerous due to the many treatments used. The prognosis relies on organ failure and delay to the treatment initiation. The early and permanent withdrawal of every suspected causal agent is of primary importance. Any reintroduction of a drug exposes the patient to the risk of a more severe recurrence.
